# Cardiac Rehabilitation in the Era of CRT and ARNI: A Missing Link in Heart Failure with Reduced Ejection Fraction Care

**DOI:** 10.3390/jcm14196766

**Published:** 2025-09-24

**Authors:** Oana Pătru, Silvia Luca, Dragoș Cozma, Cristina Văcărescu, Simina Crișan, Mihaela Daniela Valcovici, Mirela Vîrtosu, Adrian Sebastian Zus, Constantin-Tudor Luca, Simona Ruxanda Drăgan

**Affiliations:** 1 Cardiology Department, “Victor Babes” University of Medicine and Pharmacy, 2 Eftimie Murgu Sq., 300041 Timisoara, Romania; oana.patru@umft.ro (O.P.); silvia.luca0@student.umft.ro (S.L.); dragos.cozma@umft.ro (D.C.); cristina.vacarescu@umft.ro (C.V.); adrian.zus@umft.ro (A.S.Z.); constantin.luca@umft.ro (C.-T.L.); simona.dragan@umft.ro (S.R.D.); 2 Research Center of the Institute of Cardiovascular Diseases Timisoara, 13A Gheorghe Adam Street, 300310 Timisoara, Romania; 3 Doctoral School, “Victor Babes” University of Medicine and Pharmacy, 300041 Timisoara, Romania; daniela.cozma@umft.ro; 4 Institute of Cardiovascular Diseases Timisoara, 13A Gheorghe Adam Street, 300310 Timisoara, Romania

**Keywords:** cardiac rehabilitation, heart failure with reduced ejection fraction, cardiac resynchronization therapy, angiotensin receptor–neprilysin inhibitors, sacubitril/valsartan, exercise training, quality of life, functional capacity, advanced heart failure therapy

## Abstract

Heart failure with reduced ejection fraction (HFrEF) continues to impose a high burden of morbidity and mortality despite significant advances in pharmacologic and device-based therapy. Cardiac resynchronization therapy (CRT) and angiotensin receptor–neprilysin inhibitors (ARNIs) have independently demonstrated substantial benefits in symptoms, health-related quality of life (HRQoL), and survival. Cardiac rehabilitation (CR), incorporating structured exercise, education, and lifestyle optimization, is well established as an effective intervention in HFrEF, yet its role in the era of combined CRT and ARNI therapy remains insufficiently characterized. This literature review synthesizes current evidence on CR in HFrEF populations receiving CRT, ARNI, or both, highlighting its impact on HRQoL, exercise capacity, and functional outcomes. Across diverse study designs—including randomized trials, observational cohorts, and meta-analyses—CR consistently yielded clinically meaningful improvements in patient-reported HRQoL and objective measures such as six-minute walk distance (6MWD) and peak oxygen uptake. Data directly evaluating CR in patients concurrently receiving both CRT and ARNI are lacking; indirect evidence suggests CR is compatible with, and may add to, contemporary device and drug therapy. However, referral rates remain low, indicating an implementation gap despite strong evidence of benefit. The review underscores the importance of integrating CR into contemporary HFrEF care and identifies a pressing need for targeted prospective studies to define its role in patients receiving dual device–pharmacologic therapy.

## 1. Introduction

HFrEF continues to represent a substantial clinical and societal burden, affecting millions of individuals globally. Despite recent therapeutic advances, patients with HFrEF often experience persistent symptoms such as dyspnea, fatigue, exercise intolerance, and psychological distress [1]. These symptoms contribute to significantly impaired HRQoL, which has become an essential endpoint in the management of chronic heart failure (HF). Improving HRQoL is not only relevant from the patient perspective but is also associated with reduced hospitalizations and improved long-term prognosis [2,3,4,5]. As new interventional strategies, such as the facilitated angioplasty approach in ST-elevation myocardial infarction, continue to refine acute care paradigms [6], it is equally imperative to assess how integrative therapies like CR can enhance outcomes in chronic HF populations receiving combined device and pharmacologic therapy.

In recent years, the introduction of CRT and ARNIs has reshaped the treatment landscape for HFrEF. CRT improves cardiac efficiency by correcting electrical dyssynchrony, leading to reverse remodeling and improved functional capacity in appropriately selected patients [7,8]. ARNIs, particularly sacubitril/valsartan, have demonstrated superiority over ACE inhibitors in reducing cardiovascular mortality, lowering the rate of hospitalization for HF [9], and enhancing HRQoL metrics such as those measured by the Kansas City Cardiomyopathy Questionnaire (KCCQ) [10]. These two therapies are now central components of guideline-directed medical therapy (GDMT) for eligible patients with HFrEF [11]. While patients typically receive full GDMT (beta-blocker, RAAS inhibition with sacubitril/valsartan preferred or ACEI/ARB when not used, mineralocorticoid receptor antagonist, and SGLT2 inhibitor), we selected ARNI as the index RAAS agent, focusing on HFrEF patients with CRT who receive ARNI as part of guideline therapy [1].

CR, which includes structured exercise training, education, and behavioral interventions, has also been shown to improve exercise tolerance, psychological well-being, and HRQoL in HF patients [12,13,14]. International guidelines strongly recommend CR for stable HFrEF patients; however, referral and enrollment rates remain suboptimal. Moreover, the evidence base supporting CR has largely developed in an earlier era of HFrEF management, prior to the widespread adoption of CRT and ARNI [1]. As such, the interactions between CR and these newer therapies are not well understood, especially in patients receiving both CRT and ARNI.

Currently, there is a notable absence of prospective studies that specifically examine the effectiveness of CR in patients who are concurrently treated with both CRT and ARNI. While a number of trials and observational studies have investigated CR in patients with CRT or ARNI separately [15,16], the lack of stratified analyses or focused subgroups means that the potential additive or synergistic benefits of CR in this modern therapeutic context remain unexplored. This is a significant limitation, given that both CRT and ARNI independently influence functional capacity and HRQoL—key domains that CR is also designed to improve.

**Figure 1:**
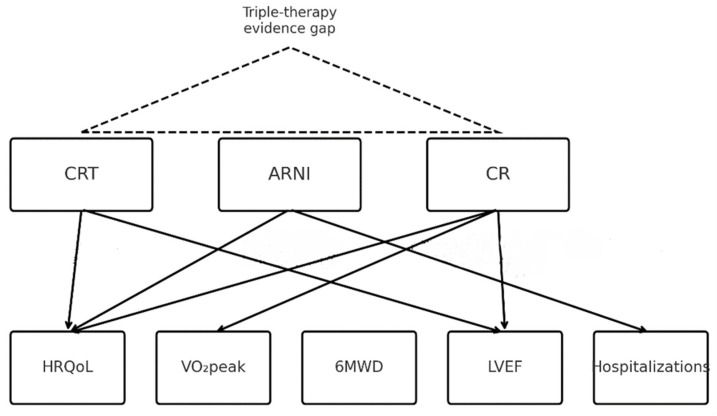
Conceptual model of CRT, ARNI, and CR effects on key outcomes in HFrEF; dashed triangle denotes triple-therapy evidence gap.

In this literature review, we aim to evaluate and synthesize the current literature regarding the impact of CR on HRQoL and functional outcomes in patients with HFrEF treated with CRT, ARNI, or both. Due to the scarcity of studies directly investigating the combined use of CRT and ARNI in the context of CR, we included studies that involve partial populations, acknowledging their methodological limitations. Our objective is to assess the consistency of reported benefits across diverse therapeutic combinations, and to identify areas of convergence, divergence, and—most importantly—gaps. Ultimately, this review seeks to underscore the urgent need for prospective, targeted studies that investigate the role of CR in this contemporary patient population, to inform evidence-based clinical recommendations and enhance individualized care strategies for those with the highest burden of disease. Figure 1 summarizes the conceptual framework underpinning this review: established effects of CRT, ARNI, and CR on HRQoL, functional capacity, and LVEF, and the persisting absence of trials evaluating concurrent CRT + ARNI + CR.

## 2. Materials and Methods

We conducted a comprehensive literature review to identify studies exploring the role of CR in patients with HFrEF treated with CRT, ARNI, or both. Our aim was to examine the relationship between CR and key outcomes—particularly HRQoL and functional capacity—in the context of contemporary HFrEF therapy. Because CR increasingly coexists with device- and drug-based care, this review also incorporated contextual evidence from CRT- or ARNI-focused studies without a CR intervention when these reported outcomes that inform how CR may perform alongside modern therapy.

**Figure 2:**
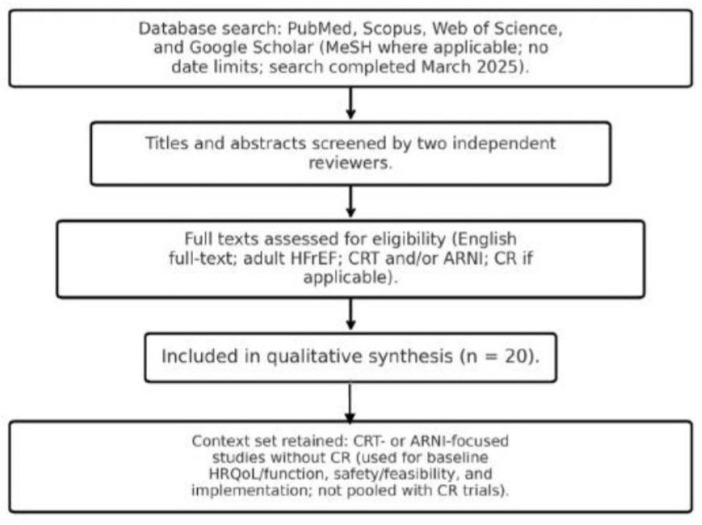
Search and selection flow for literature review.

A literature search was performed in PubMed, Scopus, Web of Science, and Google Scholar. Keyword combinations included: “cardiac rehabilitation,” “CRT,” “cardiac resynchronization therapy,” “sacubitril/valsartan,” “ARNI,” “heart failure with reduced ejection fraction,” “quality of life,” and “exercise capacity.” Where applicable, PubMed queries were refined with Medical Subject Headings (MeSH) to increase specificity. The final search was completed in March 2025. No publication-date limits were applied, to capture both historical and contemporary studies. Titles and abstracts were screened manually by independent reviewers with clinical expertise in HF care. We included peer-reviewed studies in adult patients (≥18 years) with HFrEF that tested a CR/exercise intervention in the setting of CRT, ARNI, or both, and reported at least one patient-centered outcome (e.g., HRQoL by KCCQ, Minnesota Living with Heart Failure Questionnaire (MLHFQ), or SF-36) or functional parameter (6MWD, VO_2_peak, or LVEF). To situate CR within modern care, we also retained CRT- or ARNI-focused studies without a CR intervention when they reported outcomes relevant to CR deployment and interpretation—namely, baseline HRQoL/function under contemporary therapy, safety/feasibility in device recipients (e.g., shocks during supervised exercise), or implementation metrics (e.g., CR referral at discharge). These studies were clearly labelled in tables/figures and not pooled with CR trials when summarizing CR effects. Non-English publications, pediatric or animal studies, editorials, case reports, and meeting abstracts without sufficient data were excluded. Full-text availability in English was required. The primary outcome was HRQoL assessed with validated instruments (KCCQ/KCCQ-12, MLHFQ, EQ-5D, SF-36). Secondary outcomes included functional capacity (6MWD, VO_2_peak), cardiac function (LVEF), safety (e.g., device therapies/shocks during exercise), and implementation (e.g., CR referral). The search and selection flow is summarized in Figure 2.

Screening occurred in two stages. First, titles/abstracts were assessed against eligibility criteria. Second, full texts of potentially relevant articles were reviewed to confirm inclusion and to allocate each study to the CR primary set or the context set (CRT/ARNI without CR). Data were extracted into a structured Microsoft Excel sheet (author, year, study design, population characteristics and sample size, therapy exposure and CR modality/duration where applicable, outcomes assessed, and key findings). For synthesis, studies were stratified by therapy exposure (CRT, ARNI, CR, and overlaps) and by study set (CR vs. context). Only CR-intervention studies informed narrative statements about the effects of CR on HRQoL/functional outcomes; context studies were used to describe baseline effects of CRT/ARNI, safety/feasibility in device populations, and implementation gaps (e.g., referral rates). We did not restrict inclusion to ARNI-only pharmacotherapy; studies conducted on ACEI/ARB backgrounds were retained as context when they informed CR deployment or interpretation alongside device and drug-based care. We additionally recorded age and sex descriptors/subgroup reporting when available; given heterogeneous reporting, these factors were synthesized narratively.

**Figure 3:**
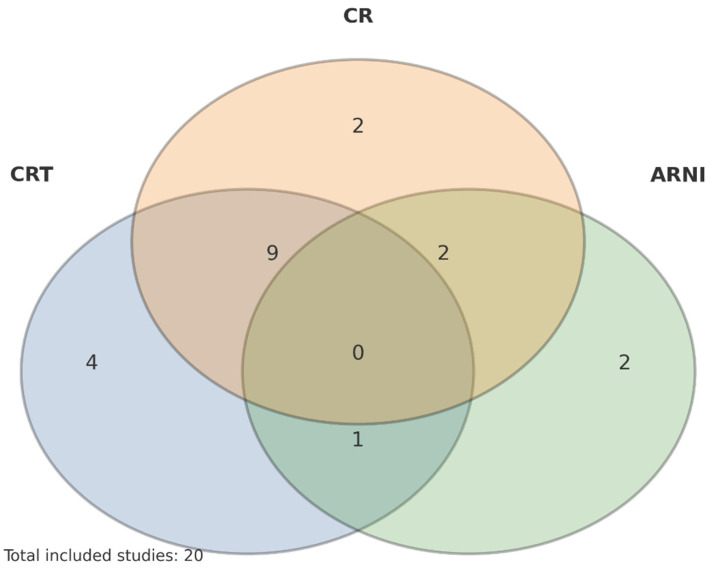
Thematic overlap of included studies by therapy exposure. Diagram illustrates how the 20 included studies relate to three core intervention categories: CRT, ARNI, and CR. No studies address all three modalities simultaneously, highlighting a critical gap in the existing literature.

Given the heterogeneity of designs, interventions, and outcome reporting, we conducted a narrative synthesis without quantitative pooling. Where appropriate, we present figures summarizing therapy overlap and timelines to map the evidence landscape and highlight gaps (notably, the paucity of trials evaluating CRT + ARNI + CR concurrently), as depicted in Figure 3. Formal risk-of-bias scoring was not undertaken; instead, study-level design features and limitations are summarized qualitatively in Section 3/Section 4.

## 3. Results

We have included in the table below the relevant articles on the subject, having specified the authors, year of publication, number of patients included, and conclusions (Table 1).

A total of 20 full-text peer-reviewed studies were included. Designs spanned randomized and nonrandomized trials, single and multicenter cohorts, registry analyses, systematic and network meta-analyses, and clinical/practice reviews. Collectively, they evaluated CR, CRT, and/or ARNI therapy in modern HFrEF care. Although most studies supported favorable effects of CR on functional capacity and health status, no study in this set prospectively tested CR in a cohort simultaneously on CRT and ARNI as a predefined population—an important evidence gap [15,16,17,18,19,20,21,22,23,24,25,26,27,28,29,30,31,32,33,34,35].

### 3.1. Characteristics of Included Studies

Study size ranged from small, focused cohorts (*n* = 18–139) to very large datasets (e.g., 289,810 discharges) [19,20,21,23,35]. By theme, CRT exposure was addressed in most publications (device-recipient trials/cohorts and meta-analyses), ARNI in several (randomized-trial synthesis, real-world effectiveness, CRT non-responder registry, and an RCT of ARNI + CR vs. ARNI alone in post-MI HFrEF) [18,22,26,33,35]. CR programs typically ran 8–24 weeks and were most often center-based supervised aerobic ± resistance training; other formats included multidisciplinary outpatient CR and telerehabilitation (e.g., remote walking guided by device/telemetry). Some cohorts reported program participation with clinical follow-up rather than a fixed “weeks” duration [21,25,27,28,30,31,34].

### 3.2. CR in Patients Receiving CRT

Across device-recipient meta-analyses and CRT cohorts, CR was consistently associated with better exercise capacity and patient-reported outcomes. A meta-analysis of RCTs in post-CRT patients found peak VO_2_ +2.17 mL/kg/min (95% CI 1.42–2.92) and LVEF +4.75% (95% CI 1.53–7.97) versus controls, with improvements in Minnesota scores [15]. A second synthesis in post-CRT HFrEF showed that light-to-moderate intensity (non-HIIT) training improved peak VO_2_ ~+3.05 mL/kg/min, LVEF ~+4.97%, and MLHFQ −19.96 points over ≤6 months, while HIIT conferred no additional benefit in that subset [31]. Ye et al. reported peak VO_2_ +2.02 mL/kg/min (95% CI 0.62–3.41) and LVEF +3.89% (95% CI 1.50–6.28) with a significant HRQoL signal (*p* = 0.028) favoring CR [27].

Prospective CRT cohorts, including responders and non-responders, showed higher peak VO_2_ after ~12 weeks of structured exercise and no exercise-related adverse events in small series [19,23]. A telemonitoring-guided walking program in CRT recipients improved EQ-5D and 6 min walk distance and reported no HF hospitalizations or deaths during the program, supporting feasibility beyond center-based models [21]. In device populations that included CRT-D, a meta-analysis found very low rates of shocks during supervised training and suggested fewer shocks over follow-up among those who exercised [32]. In older CRT recipients, HRQoL outcomes did not differ by CRT-P vs. CRT-D at 6 months, indicating device configuration per se should not limit rehabilitation enrollment [29]. Additional context from device cohorts highlights sustained HRQoL gains and increased QALYs with CRT [17], as well as heterogeneous KCCQ trajectory phenotypes that may benefit from adjunctive CR and psychosocial support [20,24].

### 3.3. CR in Patients Receiving ARNI

A randomized-trial meta-analysis showed that sacubitril/valsartan improves HRQoL (e.g., KCCQ) compared with ACEI/ARB in HFrEF, establishing a pharmacologic baseline for potential additive CR effects [26]. Real-world evidence associated ARNI initiation with lower HF and all-cause hospitalizations and reduced mortality [18]. Evidence directly testing CR layered onto ARNI is limited but informative: a single-center RCT in post-MI HF reported ARNI + CR superior to ARNI alone for peak VO_2_, anaerobic threshold, METs, and LVEF [33]. A network meta-analysis across HF populations ranked combined aerobic + resistance training as most effective for MLHFQ and 6 min walk, whereas center-based HIIT best improved LVEF, and center-based aerobic training optimized peak VO_2_—useful when tailoring CR for ARNI-treated patients [34].

### 3.4. CR in Patients Treated with Both CRT and ARNI

Within this evidence set, no study evaluated a CR program specifically in patients already on both CRT and ARNI. The RESINA prospective registry nonetheless showed that initiating sacubitril/valsartan in CRT non-responders improved KCCQ-12 and MLHFQ and reduced HF hospitalizations (without a formal CR intervention), implying that pharmacologic optimization can enhance patient-reported outcomes in difficult-to-treat device recipients [22]. A large multicenter cohort of HF patients participating in multidisciplinary outpatient CR demonstrated lower mortality and fewer HF readmissions after propensity matching, underscoring the prognostic relevance of CR participation in contemporary practice [30]. Despite these signals, a national performance analysis showed low inpatient CR referral (~10.5%) and suboptimal ARNI uptake/dosing, indicating an implementation gap in pairing guideline pharmacotherapy with rehabilitation [35].

### 3.5. Functional and QoL Outcomes

Validated instruments across studies included MLHFQ, KCCQ/KCCQ-12, EQ-5D, and SF-36. In CRT recipients, meta-analytic data showed clinically meaningful improvements with CR: VO_2_peak increases of ~2–3 mL/kg/min, LVEF gains of ~4–5%, and MLHFQ score reductions favoring CR vs. controls [15,27,31]. Cohorts reported complementary gains in 6 min walk and EQ-5D (including in tele-CR) [19,21,23]. For ARNI, randomized data confirmed better HRQoL vs. ACEI/ARB in HFrEF [26], and ARNI + CR improved exercise capacity (peak VO_2_, AT, METs) and LVEF over ARNI alone in post-MI HFrEF [33]. Safety within device recipients was favorable: supervised programs showed very low rates of arrhythmic therapies (shocks) during training, with indications of fewer shocks over follow-up among exercisers [32]. Broader device literature in the used studies supports durable HRQoL gains with CRT and highlights patient-level heterogeneity in health-status trajectories that CR may help address [17,20,24].

### 3.6. Evidence Gaps and Quality Considerations

No RCT directly evaluated CR in patients concurrently on CRT and ARNI in the uploaded set, limiting conclusions about triple-therapy synergy. Heterogeneity in CR “dose” (frequency/intensity), modality (center-based vs. home/tele), program duration, and outcome reporting precluded quantitative pooling across all studies [27,31,34]. Although several RCTs/meta-analyses demonstrate CR benefits in CRT recipients (peak VO_2_, LVEF, HRQoL) and HRQoL improvements with ARNI in HFrEF, many device cohorts were single-center and nonrandomized, and intervention details were variably reported [15,26,27,30,31]. Practice/clinical reviews provide convergent exercise-prescription guidance (aerobic ± resistance training; monitoring for device therapies), which can guide standardized protocols in future trials [25,28,32].

## 4. Discussion

This review synthesizes data from 20 peer-reviewed studies exploring the impact of CR on patients with HFrEF, including those receiving CRT or ARNI; however, data on patients already on both remain limited. While the data consistently show that CR improves functional status and HRQoL, a key limitation is the absence of prospective studies focusing specifically on patients treated concurrently with both CRT and ARNI—despite this being a growing and highly relevant clinical subgroup.

Across diverse study designs—ranging from randomized trials to meta-analyses and retrospective cohorts—CR was associated with meaningful improvements in HRQoL, exercise tolerance, and symptom burden. These benefits were observed in patients undergoing CRT, ARNI therapy, or GDMT more broadly. Improvements in validated HRQoL instruments (MLHFQ, KCCQ, SF-36) consistently exceeded minimal clinically important differences, and gains in 6MWD, peak VO_2_, and sometimes even LVEF confirmed the functional impact of CR.

### 4.1. Quality of Life: A Core Treatment Target

In modern HFrEF management, HRQoL has become a treatment target equal in importance to survival and hospitalization reduction. Many patients with HFrEF report that symptom relief and physical independence matter more to them than prolonging life. This shift toward patient-centered outcomes reinforces the centrality of CR, which is one of the few interventions shown to reliably improve both physical capacity and emotional well-being.

This review demonstrates that CR offers unique contributions beyond those of pharmacologic or device therapy alone. Even in patients already treated with CRT or sacubitril/valsartan, CR was associated with further HRQoL improvement, suggesting that these therapies are complementary rather than redundant. Importantly, studies like those by Ye et al. [27], Kabboul et al. [36], and Chen et al. [34] demonstrated additive effects when CR was layered onto other HF treatments, enhancing the patient experience of recovery.

### 4.2. Addressing the Challenge of CRT Non-Responders

A significant subset of patients undergoing CRT—estimated at 30–40%—do not respond adequately, experiencing limited or no improvement in LVEF, symptoms, or NYHA class. This phenomenon, termed CRT non-response, remains a clinical challenge with few targeted interventions [37]. The findings of this review suggest that CR may play a role in optimizing outcomes for these patients, by improving peripheral conditioning, functional independence, and even psychosocial outlook—factors not directly targeted by CRT. Additionally, recent findings by Văcărescu et al. suggest that structured exercise testing may itself serve a dual role in these patients: not only guiding rehabilitation but also revealing suboptimal device function, particularly in those with LV-only fusion pacing [38]. Building on this, Faur-Grigori et al. systematically reviewed current evidence and proposed refined patient selection criteria—such as preserved atrioventricular conduction, non-ischemic etiology, and sinus rhythm—which may predict better outcomes with LV-only fusion pacing compared to conventional biventricular CRT [39].

Although most included studies did not stratify responders vs. non-responders, observational data indicated that post-CRT patients who underwent CR still achieved clinically meaningful improvements in functional capacity and HRQoL, regardless of the degree of cardiac remodeling. This supports broader referral of CRT patients to CR programs, not only as a means of reinforcing therapy response but also as a safety net for those who fail to improve on device therapy alone.

### 4.3. Synergy Between CRT, ARNI, and CR

Patients treated with either CRT or ARNI showed consistent improvements when CR was incorporated into their care. For CRT patients, the combination of improved ventricular synchrony and structured exercise resulted in greater functional gains than CRT alone. Similarly, patients on sacubitril/valsartan benefited from the hemodynamic and neurohormonal advantages of ARNI, which, when paired with exercise, led to larger improvements in VO_2_peak, 6MWD, and patient-reported symptoms.

Across exercise modalities, network meta-analysis suggests AE/RE is most effective for improving MLHFQ and 6MWD and for reducing readmissions, while center-based HIIT most effectively improves LVEF, and center-based AE yields the greatest gains in VO_2_peak. In CRT recipients, systematic reviews/meta-analyses show clinically meaningful improvements with structured training, with moderate-intensity continuous programs consistently beneficial and no clear advantage of HIIT over non-HIIT protocols in this subgroup. In patients with implantable devices (ICD/CRT-D), supervised exercise appears safe, with very low shock rates reported during training. Taken together, these data support prioritizing AE ± RE at moderate intensity as a pragmatic default in device recipients, with HIIT reserved for experienced centers using individualized protocols and device oversight [31,34].

However, no study prospectively evaluated CR in patients already on both CRT and ARNI. Meta-analytic data support CR benefits after CRT and randomized data show ARNI improves HRQoL versus ACEI/ARB; one RCT in post-MI HFrEF found ARNI + CR superior to ARNI alone—but triple-therapy (CRT + ARNI + CR) remains untested and should be a priority for future trials [26,33]. In parallel, the trend towards personalized cardiovascular medicine—including genotype-based drug tailoring and gender-specific management—reinforces the importance of integrating individualized pharmacologic strategies with functional interventions. For example, Luca et al. highlight how targeted antihypertensive therapy can be informed by DNA sequencing, patient sex, and novel molecular markers, ultimately influencing cardiovascular outcomes in complex patients [40]. Notably, recent comprehensive analyses of drug therapy in HF—such as that by Buda et al.—have emphasized the growing complexity of pharmacologic regimens, particularly in older adults, reinforcing the importance of pairing optimized medical therapy with functional interventions like CR to maximize patient-centered outcomes [41]. Beyond HFrEF, the article by Cersosimo et al. on CR in atrial fibrillation highlights program components, potential benefits, and practical implementation considerations, underscoring the broader applicability of CR across cardiac conditions [42].

### 4.4. Gaps in Referral and Access to CR

Despite guideline recommendations, CR remains underprescribed and underutilized, even in high-risk populations such as those with CRT and ARNI. Warner et al. reported referral rates of 10.5% inpatient CR referral (with 15.0% any mention/attempt), rising over time but still low; ARNI use reached 17.2% by 2020 among eligible patients [35]. This implementation gap likely reflects a combination of structural, logistical, and educational barriers—including limited access to CR programs, poor integration into discharge planning, and underappreciation of CR’s benefits by both patients and clinicians.

### 4.5. Digital and Telerehabilitation in Contemporary HF Care

Digital and telerehabilitation have gained prominence post-pandemic as scalable strategies to overcome access barriers (distance, transport, workforce). Beyond the Koike [21] tele-walking program in CRT recipients, the TELEREH-HF [43] randomized trial (*n* ≈ 850) evaluated hybrid comprehensive telerehabilitation in HF and found clinically meaningful improvements in functional and psychological domains, although the primary composite of days alive and out of hospital was not increased versus usual care over long-term follow-up; safety and adherence were favorable. Subsequent TELEREH-HF analyses suggest benefits on psychological status (e.g., anxiety) and acceptable adherence patterns, including in subgroups such as women [44,45]. Complementary guidance and position statements in the post-COVID era endorse home-based and hybrid CR as viable, safe alternatives when appropriate supervision and telemonitoring are available [46]. Collectively, these data support implementation of hybrid models for HFrEF—including CRT/ARNI recipients who face logistical barriers—while underscoring the ongoing evidence gap for concurrent CRT + ARNI + tele-CR cohorts.

### 4.6. Frailty, Sex Differences, and Multimorbidity as Modifiers of CR Response

Frailty, multimorbidity, age, and sex-specific factors shape both eligibility and response to CR in HFrEF. Epidemiologic work shows that the lifetime risk of HFrEF is higher in men than in women and that etiologies shift with age—from genetic/idiopathic or injury-related causes in younger patients, to ischemic disease peaking in midlife, and multimorbidity predominating in older adults—implications that favor age- and sex-sensitive CR tailoring (e.g., intensity progression, resistance dosing, balance/falls prevention, cognitive and psychosocial support) [47,48]. The REHAB-HF randomized trial [49] in older, frail patients initiating a tailored, multidomain program during/after HF hospitalization significantly improved Short Physical Performance Battery scores and other functional/HRQoL measures at 3 months (without clear reductions in readmissions/mortality), demonstrating that frailty is not a contraindication to CR and that targeted prescriptions can yield meaningful patient-centered gains. Women remain under-referred and under-enrolled in CR; contemporary reviews highlight disparities across referral, participation, and outcomes, and call for sex-sensitive program design [50,51]. A recent TELEREH-HF subanalysis also supports the effectiveness of hybrid telerehabilitation in women [45]. Finally, multimorbidity (e.g., CKD, COPD, diabetes, musculoskeletal disease) is highly prevalent in HF and associates with worse function and outcomes, yet patients with multiple chronic conditions are often excluded from CR trials. Observational and narrative syntheses underscore the need to design inclusive CR programs and pre-specify stratified analyses by frailty, sex, and comorbidity burden—particularly relevant for CRT/ARNI recipients, who are frequently older and multimorbid [52].

### 4.7. Limitations of the Evidence Base and Future Directions

This review is subject to several limitations inherent in the available literature. First, no prospective study specifically designed to evaluate CR in CRT + ARNI patients was identified, limiting the ability to draw high-certainty conclusions. Second, heterogeneity in CR program structure, duration, outcome definitions, and study quality precluded formal meta-analysis. Third, many studies were observational in design, raising the potential for bias and confounding. Also, frailty, multimorbidity, and sex-specific outcomes were rarely and inconsistently reported, limiting subgroup inference to real-world CRT/ARNI populations. Finally, HRQoL tools, while validated, were not uniformly applied across studies, making direct comparisons challenging.

Despite these limitations, the review strongly supports the continued and expanded role of CR in the treatment of HFrEF—even in the era of advanced pharmacologic and device therapies.

Future research should aim to close the knowledge gap around the combined effect of CRT, ARNI, and CR in patients with HFrEF. Well-designed, prospective studies with predefined subgroup analyses, particularly distinguishing CRT responders from non-responders, are urgently needed. Standardizing CR protocols and HRQoL assessments across trials will also strengthen the interpretability and applicability of future findings.

## 5. Conclusions

CR remains one of the most consistently effective non-pharmacological interventions for improving the lives of patients with HFrEF. In this literature review of 20 studies, we found robust and repeated evidence that CR significantly enhances functional performance, exercise tolerance, and, most importantly, HRQoL. These outcomes were observed across a wide range of study designs and patient populations, including those treated with CRT and ARNIs, two of the most important pillars of modern HFrEF therapy.

Even though CRT and ARNI therapy each improve symptoms and clinical outcomes independently, our findings suggest that these benefits are not redundant with those of CR. Rather, CR appears to offer a complementary effect, further improving HRQoL and physical capacity even in patients already receiving advanced medical and device-based therapies. Notably, no study in our set prospectively tested CR in a cohort already on both CRT and ARNI; this remains a key evidence gap. This review also draws attention to an often-overlooked subgroup: CRT non-responders, who represent up to 40% of patients undergoing device implantation. While these individuals may not experience substantial reverse remodeling or hemodynamic improvement, CR appears to offer an alternative path to recovery by targeting peripheral adaptation, functional strength, and psychological resilience. For these patients, CR may be the only intervention that provides noticeable improvements in their daily living experience and self-reported well-being.

Despite its proven value, CR remains severely underutilized, particularly among patients who might benefit most. Systemic barriers, lack of standardized referral processes, and insufficient awareness among providers continue to limit CR uptake. As Warner et al. demonstrated, inpatient CR referral was ~10.5% (15.0% any mention/attempt), and ARNI use reached ~17.2% by 2020, highlighting persistent implementation gaps [35]. This represents not only a care gap, but a missed opportunity to optimize the overall treatment impact for patients with complex, high-burden disease.

Furthermore, while the evidence base is compelling, it remains incomplete. The absence of randomized controlled trials focusing specifically on patients treated concurrently with both CRT and ARNI remains a glaring limitation. As this combination becomes increasingly common in guideline-directed management, the need for prospective, stratified studies assessing CR outcomes in this dual-therapy subgroup becomes both scientifically and ethically urgent.

In conclusion, CR should not be viewed as ancillary to modern HFrEF therapy but rather as a core, essential component of multidimensional care. Its consistent ability to improve functional outcomes and enhance HRQoL makes it uniquely suited to address patient-centered goals—especially in populations where standard therapies may plateau. Efforts to integrate CR more fully into routine care, supported by updated referral protocols and expanded program access, are imperative. Looking forward, high-quality studies that evaluate the impact of CR in CRT + ARNI patients specifically will be critical to closing the remaining gaps and realizing the full potential of rehabilitation in contemporary HFrEF care.

## Figures and Tables

**Table 1:** Summary of all included studies (*n* = 20). “CR Type” indicates whether an exercise-based CR intervention was tested. Rows with “—” under CR Type are context studies (CRT- or ARNI-focused without a CR program) retained to provide baseline effects (HRQoL/function under modern therapy), safety/feasibility in device recipients, or implementation metrics; these studies were not pooled with CR trials when summarizing CR effects. Duration is reported in weeks unless noted “program-based” (phase II/III outpatient CR with variable length). Sample size (N) reflects analyzed population. Device type is listed when available (CRT-P/CRT-D). HRQoL instruments and directionality: KCCQ/KCCQ-12 (higher = better), MLHFQ (lower = better), EQ-5D index (higher = better), SF-36 domains (higher = better). Abbreviations: AE, aerobic exercise; RE, resistance exercise; VO_2_peak, peak oxygen uptake; HIIT, high-intensity interval training; AEs, adverse event; NR, not reported; -, not applicable/no CR.

Authors	Year	Sample Size (N)	Design	CRT	ARNI	CR Type	Duration (Weeks)	HRQoL Tool(s)/Key Findings
Cleland JGF et al. [17]	2009	813	Randomized trial	Yes	No	-	Median 29.6 months; HRQoL assessed at baseline, 3 months, 18 months, and study end	MLHFQ; EQ-5D—sustained HRQoL gains; ↑QALYs
Proudfoot C et al. [18]	2021	68 studies	Systematic review (real-world ARNI)	Some	Yes	-	1–19 months	NYHA; biomarkers; some KCCQ/MLHFQ—↓HF/AC hospitalizations; ↓mortality
Misumi K et al. [19]	2022	64	Observational Cohort	Yes	GDMT background	Center-based exercise CR	≈12	Peak VO_2_ primary—↑capacity regardless of CRT response
Mastenbroek MH et al. [20]	2015	139	Prospective cohort (KCCQ trajectories after CRT-D)	Yes (CRT-D)	No	-	≈56	KCCQ—distinct health-status trajectories; supports adjunctive rehab/psych care
Koike A et al. [21]	2022	18	Single-group pre–post (tele-monitored walking)	Yes	N/R	Telerehabilitation (walking)	12	EQ-5D; 6MWD—↑HRQoL and activity; safe
Chen ZB et al. [15]	2019	4 RCTs; N = 157	Meta-analysis of RCTs (CR after CRT)	Yes	N/R	Supervised exercise CR	Varied	MLHFQ; ↑peak VO_2_ (~+2.2 mL/kg/min); ↑LVEF (~+4.8%)
Rubio Campal JM et al. (RESINA) [22]	2021	35	Prospective registry (CRT non-responders → ARNI)	Yes (non-responders)	Yes	-	≈24	KCCQ-12; MLHFQ—HRQoL improved; ↓hospitalizations
Yanagi H et al. [23]	2019	34	Cohort (responders vs. non-responders)	Yes	N/R	Center-based exercise training	12	Peak VO_2_; strength—improved in both groups; no exercise-related AEs
Dixit NK et al. [24]	2010	20	Pilot pre–post (neurocognitive and HRQoL after CRT)	Yes	No	-	≈12	MLHFQ; LVD-36—improved attention/processing; ↑QoL
Haennel RG [25]	2012	-	Practice review (exercise rehab in device pts)	Yes	No	Guidance (aerobic/resistance)	-	Summarizes safe prescriptions; expected functional benefits
Song Y et al. [26]	2022	10RCTs	Systematic review and meta-analysis (ARNI vs. ACEI/ARB)	N/A	Yes	-	Varied	KCCQ/MLHFQ—ARNI improved HRQoL vs. ACEI/ARB in HFrEF
Ye L et al. [27]	2020	7 studies; 661	Systematic review and meta-analysis (CRT recipients)	Yes	N/R	Exercise rehabilitation (supervised)	8–24	MLHFQ subset—↑peak VO_2_, ↑exercise duration, ↑LVEF; no ↑SAEs
Tedjasukmana D et al. [28]	2021	-	Clinical review (exercise prescription in CRT)	Yes	-	Guidance for aerobic Rx	-	Summarizes safe, effective dosing; ICD shock considerations
Dhande M et al. [29]	2023	101	Prospective cohort (≥75 y; CRT-P vs. CRT-D)	Yes	GDMT background	-	24	SF-36; MLHFQ—no adjusted HRQoL difference between device types
Kamiya K et al. [30]	2020	3277 (matched 796 pairs)	Multicenter retrospective cohort (propensity-matched)	Some	No	Multidisciplinary CR (phase II)	Program-based	CR participation ↓all-cause mortality and ↓HF readmissions (propensity-matched)
Guo R et al. [31]	2021	7 RCTs; 235	Systematic review and meta-analysis (post-CRT CHF)	Yes	N/R	Exercise training; non-HIT vs. HIIT	≤24	MLHFQ; peak VO_2_; LVEF—non-HIIT improved VO_2_, LVEF, HRQoL; HIIT no advantage
Steinhaus DA et al. [32]	2019	16 studies; 2547	Systematic review and meta-analysis (ICD/CRT-D)	CRT-D subset	-	Exercise interventions	3–24	Safety—very low shocks during training; fewer shocks with exercise over follow-up
Zhao Y-M et al. [33]	2025	118	Single-center RCT (post-AMI HF)	No	Yes (both arms)	CR added to ARNI	Unspecified	CPET; LVEF—ARNI + CR superior to ARNI alone for VO_2_peak, AT, METs, LVEF
Hua C et al. [34]	2024	33 RCTs	Network meta-analysis (CR modalities in CHF)	Mixed/unspecified	Unspecified	Center/home/tele; AE/RE/HIIT	Varied	MLHFQ; 6MWD; LVEF—AE + RE best for HRQoL/6MWD; CB-HIIT best for LVEF; CB-AE best for peak VO_2_
Warner AL et al. [35]	2022	289,810 HF discharges	Retrospective cohort (performance measures)	Devices tracked	Eligible 68%; use up to ~17% by 2020	CR referral metric	-	R referral low (~10.5%); ARNI uptake/dosing suboptimal; wide site variability

↓—decreased; ↑—increased.

## Data Availability

No new data were created or analyzed in this study. Data sharing is not applicable to this article.
